# Development of Hollow Steel Ball Macro-Encapsulated PCM for Thermal Energy Storage Concrete

**DOI:** 10.3390/ma9010059

**Published:** 2016-01-19

**Authors:** Zhijun Dong, Hongzhi Cui, Waiching Tang, Dazhu Chen, Haibo Wen

**Affiliations:** 1 Guangdong Provincial Key Laboratory of Durability for Marine Civil Engineering, College of Civil Engineering, Shenzhen University, Shenzhen 518060, China; dongzj@sziit.edu.cn (Z.D.); dzchen@szu.edu.cn (D.C.); haibowen2-c@my.cityu.edu.hk (H.W.); 2 School of Architecture and Built Environment, University of Newcastle, Callaghan, NSW 2308, Australia; patrick.tang@newcastle.edu.au

**Keywords:** phase change materials, hollow steel ball, macro-encapsulated, thermal properties, mechanical properties, structural-functional integrated concrete

## Abstract

The application of thermal energy storage with phase change materials (PCMs) for energy efficiency of buildings grew rapidly in the last few years. In this research, octadecane paraffin was served as a PCM, and a structural concrete with the function of indoor temperature control was developed by using a macro-encapsulated PCM hollow steel ball (HSB). The macro-encapsulated PCM-HSB was prepared by incorporation of octadecane into HSBs through vacuum impregnation. Test results showed that the maximum percentage of octadecane carried by HSBs was 80.3% by mass. The macro-encapsulated PCM-HSB has a latent heat storage capacity as high as 200.5 J/g. The compressive strength of concrete with macro-encapsulated PCM-HSB at 28 days ranged from 22 to 40 MPa. The indoor thermal performance test revealed that concrete with macro-encapsulated octadecane-HSB was capable of reducing the peak indoor air temperature and the fluctuation of indoor temperature. It can be very effective in transferring the heating and cooling loads away from the peak demand times.

## 1. Introduction

The increase in energy demand, shortage of fossil fuels and environmental concerns have provided a driving force to the development of sustainable building and renewable energy resources [1]. Direct solar radiation is believed to be one of the most potential energy sources [2]. However, it is known that solar energy is intermittent and its utilization requires proper and efficient energy storage system [3]. Latent heat storage utilizing organic phase change materials (PCM) has attracted researchers and engineers due to its numerous advantages, including high energy storage density, small temperature change from storage to retrieval, chemically inert state, high thermal stability, lack of segregation, non-supercooling quality, and non-corrosive and non-toxic properties [4]. In this research, an organic PCM, octadecane paraffin, was used as a phase change material. 

In order to utilize PCM conveniently, PCM can be encapsulated using different methods, such as micro-encapsulation or macro-encapsulation [5,6,7,8,9,10,11,12]. Alkan *et al*. [13] prepared a micro-encapsulated PCM for thermal energy storage. They used docosane and polymethylmethacrylate as core and shell materials for the micro-encapsulated PCM, respectively. Arce *et al*. [14] carried out a study about effects of PCM on comfort conditions inside the building, and compared them to those obtained without employing awnings. The south, west and roof walls of the test room models were cast using concrete containing about 5% in weight of micro-encapsulated PCM (Micronal^®^ PCM, from BASF, Ludwigshafen, Germany) with a theoretical melting point of 26 °C, and a phase change enthalpy of 110 kJ/kg). One issue of micro-encapsulated PCM is that the organic shell material can hinder the heat transfer and reduce the efficiency of PCM thermal energy storage.

In macro-encapsulation method, PCM stored in containers (tubes, spheres, panels, *etc*.) can be incorporated into building elements without affecting the function of the building [15,16]. Shi *et al*. [16] used a steel box to encapsulate PCM and assessed the effect of different positions of macro-encapsulated phase change material in concrete walls on indoor temperatures and humidity levels. Cui and Memon [15,17] developed thermal energy storage concrete by incorporating PCM in porous lightweight aggregates (LWAs). Thermal energy storage aggregates were prepared with a vacuum impregnation technique. It was found that porous aggregates and PCM are chemically compatible and have large thermal energy storage density. However, the process of adding graphite powder for increasing thermal conductivity of sealing materials is complex and may result in low work efficiency. 

Based on the abovementioned current situation of PCM applications, it is necessary to develop a simple and efficient PCM encapsulation method for the production of structural-functional integrated concrete. It is believed that a hollow steel ball (HSB) can be served as coarse aggregate and the steel has excellent thermal conductivity compared with organic materials and cement based materials. Therefore, in this study, a macro-encapsulated PCM-HSB was prepared and used in normal weight aggregate concrete (NWAC) to develop structurally–functional integrated concrete. The thermal properties of PCM-HSB, thermal performance of PCM-HSB concrete and mechanical properties of the concrete were investigated.

## 2. Results and Discussion

### 2.1. Thermal Properties and Reliability of PCM-HSB

**Figure 1:**
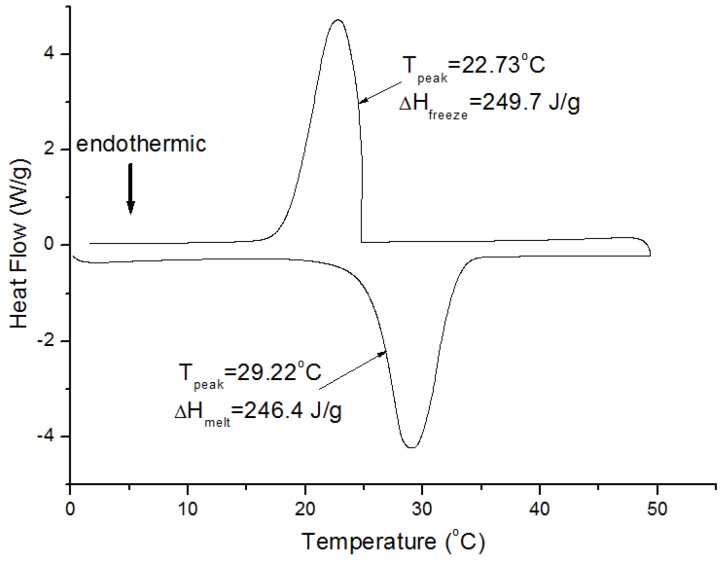
DSC curve of octadecane.

From the Differential Scanning Calorimetry (DSC) curve of PCM in Figure 1, the phase-change temperature ranges of octadecane are approximately 21–33 °C for the melting process and 18–25 °C for the solidification process. Moreover, the peak melting temperature and enthalpy are determined to be 29.2 °C and 246.4 J/g for the fusion process of PCM and 22.7 °C and 249.7 J/g for the freezing process, respectively. The mean enthalpy of octadecane is about 248.1 J/g. Kheradmand *et al*. [18] studied the effect of different heating/cooling rates (0.1 °C/min, 1 °C/min, 2 °C/min, 4 °C/min, 6 °C/min) on the DSC test results. Their results showed that, although the overall differences in the thermograms for each rate heating/cooling are smaller as the rate decreases, the accumulated specific enthalpy is almost constant regardless of the heating/cooling rate. Therefore, in this research, the rate of heating and cooling for the DSC tests was 5 °C/min. The absorption ratio of PCM to HSB is 80.3 wt% based on the vacuum impregnation test. Then, the latent heat of PCM-HSB can be determined to be about 199.2 J/g (248.1 × 80.3% = 199.2). Compared with the enthalpies between 15 J/g and 150 J/g, as reported in other PCM research [15,17,19,20], the latent heat of PCM-HSB is considered relatively high. For a given mass, the higher the enthalpy of PCM, the greater temperature controlling ability the PCM is; therefore, it is believed that PCM-HSB is one of the good candidates for thermal energy storage in building applications.

The thermal reliability of the PCM-HSB was evaluated by measuring the leakage percentage of PCM-HSB after the thermal cycle tests. The leakage percentage of PCM-HSB was calculated based on the change of mass using the following Equation (1).

**Figure 2:**
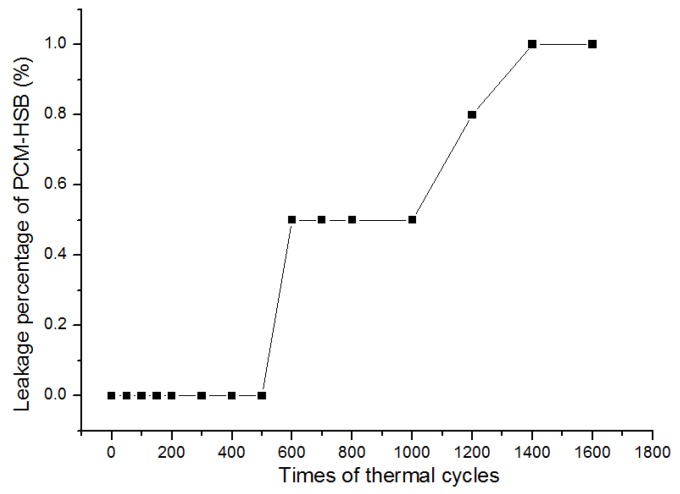
PCM-HSB leakage percentage under different times of thermal cycles.

Leakage percentage of PCM-HSB (wt%) = ΔM_PCM_/M_PCM-HSB_ × 100% (1)

where ΔM_PCM_ represents the mass loss of PCM under a certain number of thermal cycles; M_PCM-HSB_ is original average mass of the 100 PCM-HSB samples. Figure 2 presents the leakage percentage under different thermal cycles. The figure clearly shows that there was no mass loss (or no leakage) that was observed for PCM-HSB up to 500 thermal cycles. When the thermal cycles increased to 1000, there was only 0.5% leakage percentage noted. The leakage percentage of PCM-HSB was less than 1% after 1600 thermal cycles. The results show that the thermal integrity and reliability of PCM-HSB is very high and the PCM-HSB is believed to have a great potential for long-term application in building.

### 2.2. Thermal Performance of PCM-HSB Concrete

The thermal performance of the NWAC panel with and without macro-encapsulated paraffin-HSB was evaluated by monitoring the temperature variation at the inner and outer surfaces of the concrete panel and at the centre of the room during the 11 h test period. The results of maximum temperature observed at different measuring positions are shown in Table 1. As indicated in Table 1, the maximum temperature value observed at the outer surface of the panel made of control concrete was the highest (55.3 °C), and the difference in temperature between the outer surface of the panel and the center of the room was also the highest (14.1 °C). The indoor temperature values observed for PCM-HSB panels were significantly lower than those observed for a concrete panel by a range of 25% to 33%. These findings clearly indicate that the PCM-HSB concrete panel was capable of absorbing a heating load significantly and reducing the peak indoor air temperature. As shown in Table 1, the peak temperature values observed at the outer surface of all concrete panels with and without PCM-HSB occurred at the same time (*i.e.*, 15:00). However, the test rooms with PCM-HSB concrete panel gave delayed response in temperature change with respect to the control model, and the significance increased with the proportion of PCM-HSB in the concrete panel. 

**Figure 3:**
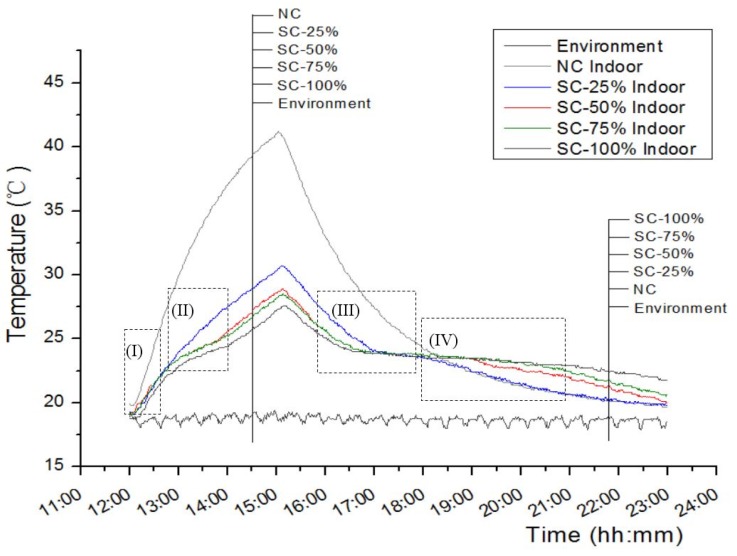
Indoor temperature curves of test room models with five different PCM-HSB panels.

Figure 3 shows the temperature variation curves of the room models with the concrete panel prepared with different proportions of macro-encapsulated PCM-HSB. Basically, the indoor temperature trends over time were the same; however, the peak values and amplitude of fluctuations were different. For the sake of convenience in further analysis, the curve is divided into four areas as indicated in the same figure. In area (I), where the heating was in progress, it can be observed that the rooms with PCM-HSB panels showed a lower rate of indoor temperature increase during the heating process when compared to the control room model. It can be deduced that part of the heating load has been taken by the paraffin macro-encapsulated in the HSB when it started to melt. In area (II), the differences in indoor temperature values between concrete room models and rooms with PCM-HSB panels became very obvious. Apparently, the indoor temperature values were reduced with the increase in PCM-HSB content. The peak indoor temperature values were 41.2 °C for the control room and 27.6 to 30.7 °C for the PCM-HSB rooms. The temperature curves for rooms with concrete panels made of SC-50% and SC-75% are very similar and they showed lower indoor temperature than that of rooms with SC-25% concrete panel; however, the rooms with SC-100% concrete panels showed the lowest indoor peak temperature. After the heating was removed, all the room models were left to cool down close to an environmental temperature of about 18 °C. As shown in Figure 3, the room with control panels cooled at a faster rate than rooms with PCM-HSB panels; however, their indoor temperature values were still higher than those of other rooms up to 18:00. In area (III), it can be seen that the rate of cooling observed for rooms with PCM-HSB panels declined gradually and dropped to nearly zero. It is believed that PCM in HSB started to solidify and release heat, so that the rate of cooling was reduced and the indoor temperature was maintained. This thermal function became obvious after 18:00. As shown in area (IV), it can be seen that the rooms with PCM-HSB panels showed higher indoor temperature than that of rooms with control panels, and the significance increased with the proportion of PCM-HSB in the panel. This result clearly shows that concrete panels with PCM-HSB are helpful in reducing indoor temperature fluctuations. Therefore, it can be concluded that PCM-HSB concrete has a function of reducing energy consumption by decreasing the temperature and shifting the loads away from the peak periods, therefore presenting promising applications in building materials.

**Table 1:** Thermal properties of room model using different phase change material–hollow steel ball (PCM-HSB) concrete panels.

Sample No.	The Maximum Temperature (°C)			Maximum Temperate Difference	Time of Peak Temperature (Delay Time)		
	OS	IS	Indoor		OS	IS	Indoor
NC	55.3	50.7	41.2	14.1	15:00	15:01(+0:01)	15:01(+0:01)
SC-25%	39.0	36.2	30.7	8.3	15:01	15:05(+0:04)	15:06(+0:05)
SC-50%	36.9	32.9	28.9	8.0	14:59	15:04(+0:05)	15:06(+0:07)
SC-75%	36.4	32.5	28.5	7.9	15:01	15:07(+0:06)	15:09(+0:08)
SC-100%	35.1	30.7	27.6	7.5	15:00	15:07(+0:07)	15:09(+0:09)

Notes: OS = outer surface; IS = inner surface; Indoor refers the temperature at the center of the room.

### 2.3. Mechanical Properties

The 28 d compressive strengths of the concretes with different PCM-HSB contents are presented in Table 2. From the Table, it can be seen that the compressive strength of PCM-HSB concrete decreased with an increase of PCM-HSB content in the concrete mix. For SC-25% concrete with just 25% PCM-HSB content in the mix, its compressive strength was found to be decreased by nearly 20% compared to the control concrete. When the PCM-HSB replacement ratio in the mix was 100%, the concrete strength was reduced significantly by 55%. The reduced compressive strength is mainly due to the smooth surface of PCM-HSB that weakened the bond at the interfacial zone between coarse aggregate and concrete matrix. 

**Table 2:** 28d compressive strength of PCM-HSB concrete.

Sample No.	28 d Compressive Strength (MPa)	Reduction of Compressive Strength in Respect to the Control (%)
NC (control)	48.8	-
SC-25%	39.7	19%
SC-50%	33.8	31%
SC-75%	29.6	39%
SC-100%	22.0	55%

**Figure 4:**
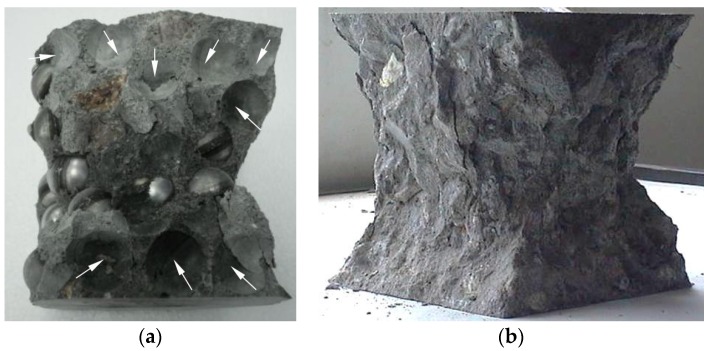
Typical failure patterns of concrete cube (**a**) PCM-HSB concrete and (**b**) NWAC concrete.

Figure 4 shows the typical failure pattern of PCM-HSB concrete cube compared with NWAC cube. In Figure 4a, the white arrows indicate the interface between PCM-HSB and mortar matrix after failure. From the Figure, it can be seen that the interface between PCM-HSB and cement paste matrix is very smooth. On a contrary, there was a sound interlocking between normal coarse aggregate and matrix in NWAC as shown in Figure 4b. Compared with the failure pattern of NWAC, for PCM-HSB concrete, the effect of interface between aggregate and matrix on concrete strength is more remarkable. This kind of smooth interface can reduce the mechanical bonding between coarse and cement paste matrix [21]. The poor mechanical bonding would lead to the decrease of compressive strength of PCM-HSB concrete. To address the poor interface of granule and mortar on the mechanical strength of the concrete, researchers have utilized different surface treatments. Zhang *et al*. [22] used a silane coupling agent and binder to modify the surface of the microcapsule in order to improve the compatibility between the microcapsule and inorganic cementing materials, thus enhancing the compressive strength of the cement mortar with smooth microcapsules. In addition, Memon *et al*. [17,23] used silica fume to eliminate the adverse effect of smooth surface of coarse aggregate on concrete compressive strength. In further studies of PCM-HSB concrete, the bonding between PCM-HSB and the matrix can be enhanced by adding superfine mineral admixture and/or increasing the surface roughness of HSB. 

Despite the compressive strength being considerably reduced with the content of PCM-HSB in the mix, the compressive strength of SC-100% concrete in this research was found to be as high as 22 MPa, which has satisfied the minimum compressive strength requirement of 15 MPa for structural application according to ACI 301-96 [24].

## 3. Materials and Methods

### 3.1. Materials Required for the Development of Macro-Encapsulated PCM-HSB

Paraffin octadecane procured from China (Sinopec Group, Lanzhou, China) was used as the PCM. Table 3 shows the basic properties of octadecane.

**Table 3:** Basic properties of octadecane.

Appearance	Relative Density (g/cm^3^)	Melting Temperature (°C)	Latent Heat Thermal Energy (J/g)
Colorless liquid	0.78	27.1	249.7

**Figure 5:**
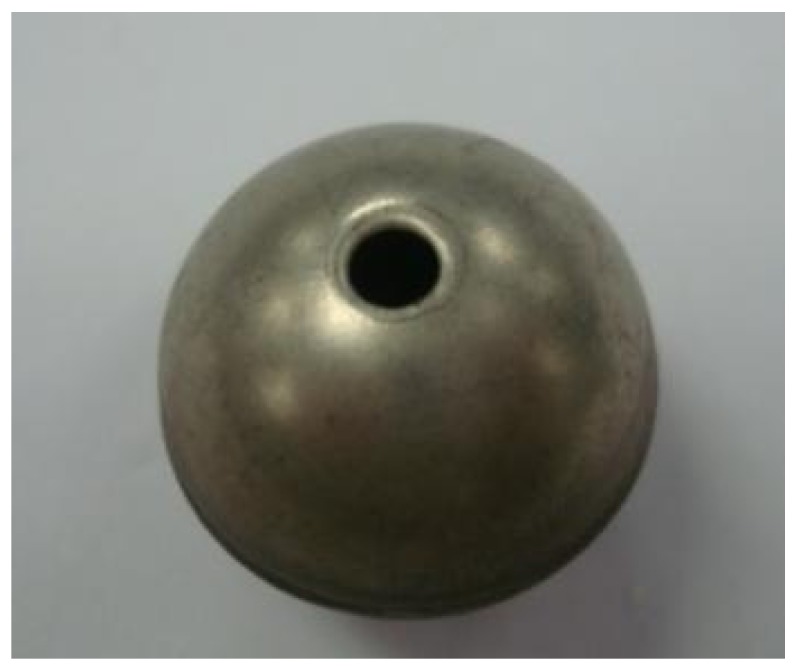
Hollow steel ball with hole.

A hollow steel ball with a nominal size of 22 mm (Figure 5) manufactured from a local supplier (Pengcheng Steelwork Co. Ltd., Shenzhen, China) was used as a coarse aggregate and container for the PCM. Each hollow steel ball is formed by welding two steel hemispheres together. The basic properties of HSB are listed in Table 4. Each HSB has a circular hole with a diameter of 2.7 mm that could allow the PCM to be stored inside the HSB during the vacuum impregnation process. 

**Table 4:** Dimension of hollow steel ball.

Inner Diameter (mm)	Outer Diameter (mm)	Hole Diameter (mm)	Apparent Density (kg/m^3^)
20.6	22	2.7	1039

### 3.2. Preparation of Macro-Encapsulated PCM-HSB

**Figure 6:**
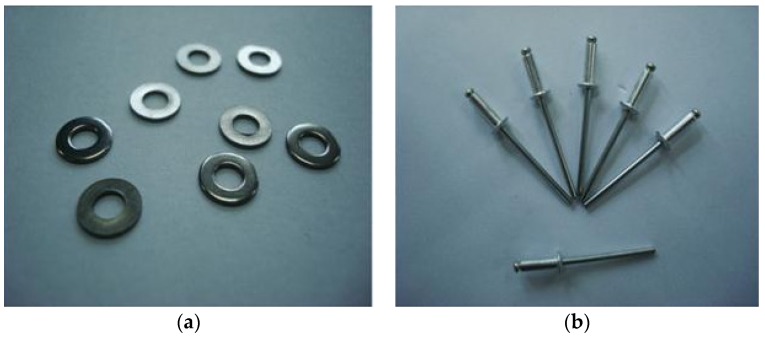
(**a**)Washer and (**b**) rivet.

**Figure 7:**
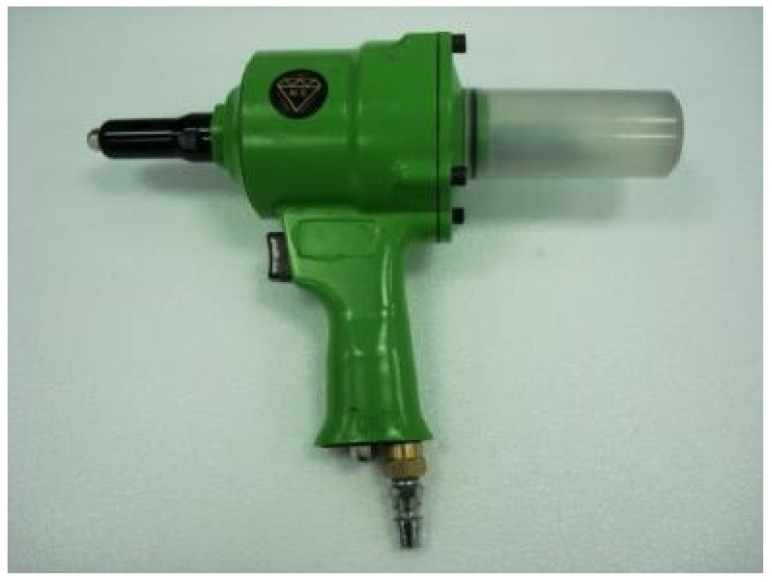
Pneumatic rivet gun.

**Figure 8:**
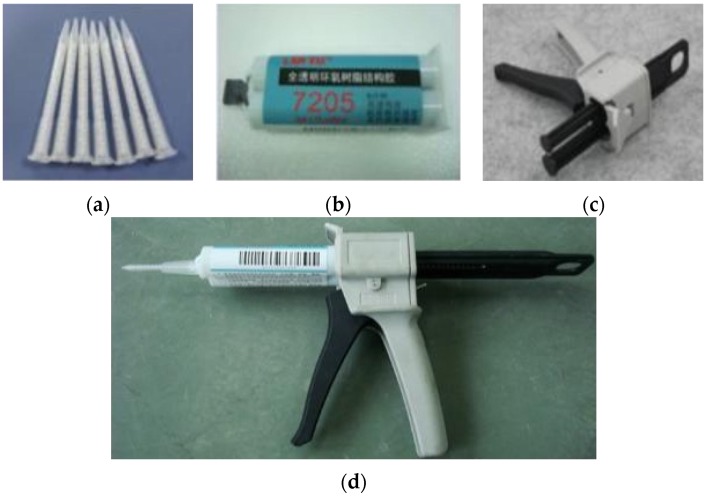
Glue gun configuration: (**a**) plastic nozzle; (**b**) epoxy; (**c**) glue gun; (**d**) complete setup.

**Figure 9:**
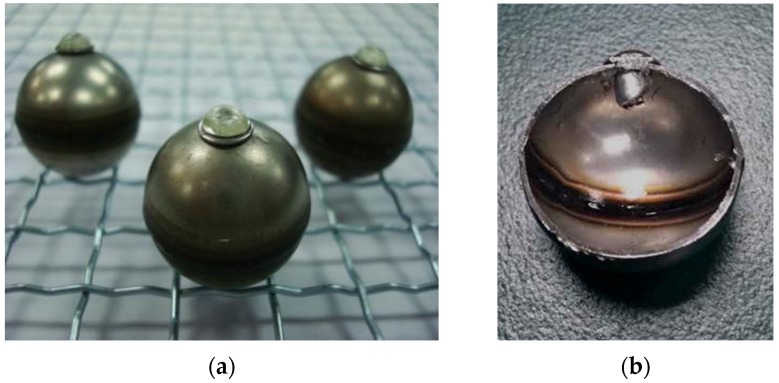
Secured paraffin-HSB: (**a**) secured with rivet and epoxy; (**b**) section of HSB.

The PCM-HSB was prepared using the vacuum impregnation setup shown similar to the previous study [18]. Initially, the HSB and melted PCM were added to a beaker placed inside the vacuum chamber. Subsequently, the sample was vacuumed at a pressure of −0.1 MPa until there were no air bubbles coming from the HSB through the circular hole. The vacuum time was about 20 min. Then, the octadecane-HSB sample was removed and each hole of HSB was fastened with stainless steel rivets (2.4 mm diameter and 6 mm in length) and washers (see Figure 6) using a pneumatic rivet gun as shown in Figure 7. The size of the washer was 2.5 mm × 6 mm × 0.5 mm (internal diameter × external diameter × thickness). To prevent leakage of octadecane from the hole, epoxy resin was used to seal the rivet by a glue gun as shown in Figure 8. Finally, the surface of paraffin-HSB was cleaned with a dry paper towel. Figure 9 shows the typical appearance of sealed paraffin-HSB. The percentage of paraffin octadecane retained by the HSB was 80.3% by mass of the hollow steel ball. The calculated apparent density of sealed octadecane-HSB was 1670 kg/m^3^.

### 3.3. Test Method for the Characterization of Macro-Encapsulated PCM-HSB

#### 3.3.1. Thermal Capacity of PCM-HSB

The phase-change behaviour of octadecane used in this research, including its phase-change temperature and latent heat, was determined using differential scanning calorimetry (DSC, DSC-Q200, TA Instruments, New Castle, DE, USA). The temperature accuracy is ±0.1 °C, and the calorimetric reproducibility (indium metal) is ±0.1%. The test was performed under a nitrogen atmosphere (40 mL/min), and the samples were tested in a temperature range of 0–60 °C at 5 °C/min heating/cooling rate. The thermal properties were determined by processing the test results with TA software Universal Analysis 2000 (TA Instruments-Waters LLC, Shanghai, China). Cui *et al*. [25] have studied the thermal properties of octadecane PCM previously, and the results showed that the energy storage capacity of PCM did not reduce after the 10 thermal cycles. Therefore, in this study, only one PCM sample and one repetition was tested in the DSC study. 

Based on the latent heat of the octadecane and the maximum percentage of octadecane carried by HSBs, the latent heat storage capacity of macro-encapsulated PCM-HSB can be obtained.

#### 3.3.2. Thermal Cycle Test

The thermal integrity of the macro-encapsulated PCM-HSB was evaluated with respect to the change in its weight after 1600 cycles of melting and freezing. This process was performed by maintaining the sample in a temperature and humidity programmable chamber (model No. BE-TH-150H3) manufactured by Dongguan Bell (Dongguan, China). The temperature range of each thermal cycle changed from 10 to 50 °C and then back to 10 °C in 1 h. The thermal cycle started when the sample was heated from 10 to 50 °C in 20 min and maintained at 50 °C for 10 min. Then, the temperature was reduced from 50 to 10 °C in 20 min, and the temperature was held at 10 °C for 10 min, and then the cycle ended. A total of 100 PCM-HSB samples were weighed before and after the thermal cycles. In this research, any mass loss of PCM-HSB was measured after 50, 100, 150, 200, 300, 400, 500, 600, 700, 800, 1000, 1200, 1400, and 1600 thermal cycles. Then the leakage percentage of PCM-HSB was calculated.

### 3.4. Materials and Mix Proportion for Concrete with PCM-HSB

Ordinary Portland cement that complied with GB 175-2007 (common portland cement) [26] was used in all of the mixes. River sand complying with the requirements of ISO R679 (methods of testing cements—determinations of strength) [27] was used as fine aggregate. The density and fineness modulus of the fine aggregate were 2600 kg/m^3^ and 2.67, respectively. Crushed granite complying with the requirements of BS 882:1992 (specification for aggregates from natural sources for concrete) [28] and having a density of 2600 kg/m^3^ was used as the normal weight coarse aggregate. Table 5 shows the physical properties of the coarse aggregate and PCM-HSB. All mixes were designed for a constant water cement ratio of 0.35. Moreover, a locally available third generation superplasticiser (polycarboxylate ether, BASF, Guangzhou, China) was used to achieve the required workability in the concrete mixes. The maximum dosage used was 1% by mass of cementitious materials. 

**Table 5:** Basic properties of coarse aggregate and PCM-HSB.

Aggregate Type	Apparent Density (kg/m^3^)	Size (mm)
Coarse Aggregate	2600	14–26
PCM-HSB	1670	22

In this research, a control concrete (NC) and four concrete mixes with different PCM-HSB contents were studied. These four PCM mixes were proportioned by replacing 25, 50, 75 and 100 percent of gravel normal coarse aggregate, which conformed to BS 882:1992 (specification for aggregates from natural sources for concrete), from the control concrete with an equal bulk volume of PCM-HSB respectively. The details of the mix proportions of control concrete and PCM-HSB concrete are given in Table 6. The admixture used in this study is polycarboxylic superplasticizer. For SC-xx%, SC means steel-ball concrete, xx is the replaced percentage of normal coarse aggregate by PCM-HSB.

**Table 6:** Mix proportion of concrete (1 m^3^ concrete).

Type	Cement (kg)	Water (kg)	Sand (kg)	Gravel (kg)	PCM-HSB (kg)	Admixture (kg)
NC	400	140	787	1092	0	4
SC-25%	400	140	787	819	175	4
SC-50%	400	140	787	546	351	4
SC-75%	400	140	787	273	526	3.5
SC-100%	400	140	787	0	701	3.5

### 3.5. Concrete Mixing

For the NC mixes without PCM-HSB, all of the solid particles were initially mixed for 1 min to homogenize the dry components. Next, water and superplasticiser were added to the dry mixture, and mixing was continued for 2 min.

The mixing of concrete with PCM-HSB was performed in several steps. Initially, all of the solid particles, excluding the PCM-HSB, were mixed for 1 min. Then, water and superplasticiser were added to the dry mixture, and mixing was continued for 1 min. Finally, PCM-HSB was added, and mixing was continued for an additional 1 min. It is important to add the PCM-HSB as the last component to avoid damaging the macro-encapsulated PCM-HSB during the mixing process as a prolonged mixing time may lead to weld cracking and joint failure of HSB.

### 3.6. Test Methods for Determining the Properties of PCM-HSB Concrete

#### 3.6.1. Compressive Strength Test

The compressive strength of concrete was determined according to Chinese GB/T 50081-2002 (standard test method of mechanical properties on ordinary concrete) [29]. A loading rate of 0.5 MPa/s was applied, which was in the range 0.5–0.8 MPa/s as prescribed by the code. For each mix, three samples were used to determine the compressive strength at an age of 28 days. The final compressive strength is an average of compressive strength of the three samples. The strength was then converted to a 150 mm cube compressive strength by multiplying it by a factor of 0.95, as suggested by the code. The maximum standard deviation in compressive strength was less than 1.5 MPa.

#### 3.6.2. Thermal Performance of PCM-HSB Concrete Panel Indoor Test

**Figure 10:**
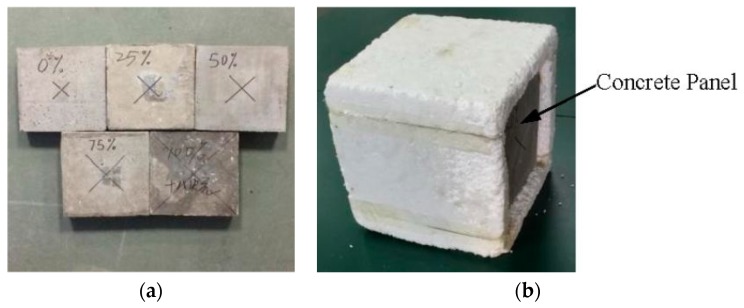
(**a**) Concrete panels with different replacement levels of coarse aggregate with PCM-HSB; (**b**) a test room model installed with a concrete panel made of SC-100%.

**Figure 11:**
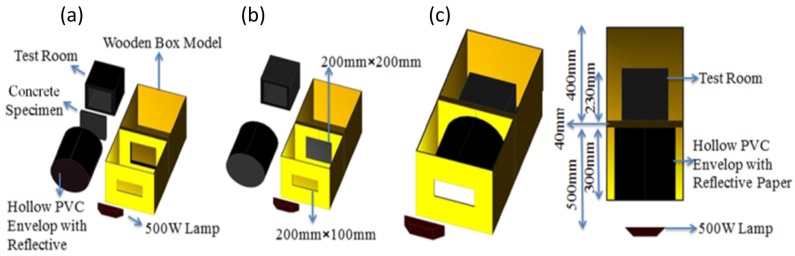
Schematic diagrams of thermal performance experiment setup: (**a**) component diagram; (**b**) installation diagram of the specimen; and (**c**) experiment setup and its top view.

The thermal performance of the PCM-HSB concrete panels with dimensions of 200 mm × 200 mm × 40 mm (Figure 10a) was evaluated at the age of 28 days using a self-designed heating system (Figure 11). The setup consisted of a wooden box model with two compartments separated by an internal wooden wall with an opening of 200 mm × 200 mm × 40 mm. A test room (made of foam with an internal dimension of 200 mm × 200 mm × 200 mm) with an opening of the same size as the internal wall opening was placed inside the compartment at the back, while a hollow PVC envelope with reflective paper coated inside to create a uniform and steady temperature field [30] was placed in the front compartment. The concrete panel was used to seal the opening of the test room as shown in Figure 10b. Appropriate sealant was used to ensure void free application of concrete panel to the test room.

**Figure 12:**
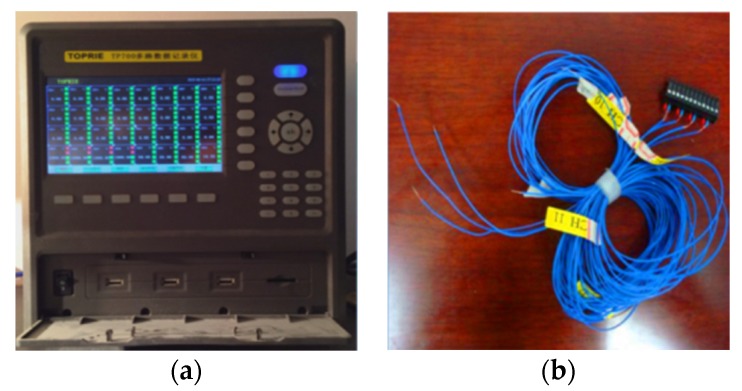
(**a**) Multi-channel data log and (**b**) K type thermal couple.

Four thermocouples (type K resolution ±0.3 °C) were used to measure the temperatures at different positions. One of them was placed at the center of the test room, and two of them were placed at the outer and inner surfaces of the concrete panel. The fourth one was placed within the compartment (where the test room was stored) to measure the environmental temperature. A 500 W lamp (used as a heating source) was placed at a distance of 500 mm in front of the test room. All the test samples were heated under the same condition and duration. The test sample was first heated up by the lamp for a period of 3 h (started at 12:00), then the heat source was removed (at 15:00) and the sample was left to cool to an environmental temperature (ended at 23:00). The temperature measurements were recorded by a data-logger as shown in Figure 12.

## 4. Conclusions

In this research, a structurally-functional integrated concrete was developed using macro-encapsulated paraffin-HSB. The use of hollow steel ball is an innovative PCM incorporation method. Based on the test results, the following conclusions can be drawn:

(1) Each HSB was found to carry up to 80.3% of PCM by mass. The leakage of PCM from the sealed HSB was found to be less than 1% after 1600 cycles of thermal test. The use of a hollow steel ball (HSB) is believed to be an effective macro-encapsulation method to carry PCM, as the thermal conductivity and reliability of the storage system can be enhanced. With such high reliability, PCM-HSB is believed to have a great potential for long-term application in building.
(2) The compressive strength of PCM-HSB concrete decreased with the increase in PCM-HSB content in the mix. The strength of SC-100% was found to be 22 MPa which is suitable for use as structural materials in construction industry.
(3) From the self-designed thermal performance setup, it can be concluded that concrete panels incorporated with macro-encapsulated PCM-HSB functions to reduce the peak indoor air temperature and the fluctuation of indoor temperature can be very effective in transferring the heating and cooling loads away from the peak demand times. The significance of these functions was increased with the increase in PCM-HSB contents. In summary, macro-encapsulated PCM-HSB can improve the thermal performance of building materials, especially during the summer, in which the temperature is well above the phase change temperature of the macro-encapsulated PCM.
